# Achieving Health Equity by Normalizing Cardiac Care

**DOI:** 10.1089/heq.2018.0067

**Published:** 2018-12-28

**Authors:** Cheryl Pegus, Ian Duncan, Judy Greener, Juan F. Granada, Tamim Ahmed

**Affiliations:** ^1^ Caluent, Inc., Philadelphia, Pennsylvania.; ^2^ Department of Statistics and Applied Probability, University of California, Santa Barbara, Santa Barbara, California.; ^3^ Inside Edge Consulting.; ^4^ Cardiovascular Research Foundation, Columbia University Medical Center, New York, New York.; ^5^ Santa Barbara Actuaries, Inc., Santa Barbara, California.

**Keywords:** acute myocardial infarction, aortic stenosis, intermediate coronary syndrome, Medicare, percutaneous transluminal coronary angioplasty, transcatheter aortic valve replacement

## Abstract

**Purpose:** It is well known that minority patients, and particularly African Americans undergo lower rates of cardiac procedures than the white population, even when covered by equivalent insurance.

**Methods:** We analyzed the rates of percutaneous transluminal coronary angioplasty (PTCA) for acute myocardial infarction (AMI) and for intermediate coronary syndrome (ICS), and rates of transcatheter aortic valve replacement for aortic stenosis in the 2012–2013 Medicare Limited Data Set (5% sample) file.

**Results:** Although blacks have similar prevalence rates for AMI and ICS, they experience lower PTCA rates when compared with that of white patients (10.57 vs. 19.40, −46%). “Normalizing” procedure rates in the African American community to match their disease prevalence will require education and participation of all stakeholders: patients, providers, manufacturers, insurers, and advocacy organizations. Beyond improved clinical outcomes, financial incentives to “normalize care” exist. We estimate “lost” revenue within the Medicare population as a result of the lower procedure rates, at ∼$90 million annually ($22.0 million AMI, $9.4 million ICS and $68.7 million aortic valve disease).

**Conclusions:** Providing evidence-based care to all patients improves health equity and can lower downstream high-cost conditions such as heart failure and multiple repeat inpatient admissions. As we move toward value-based care, the opportunity to normalize treatment for everyone seeking care is within our data analytics, innovative and collective reach.

## Introduction

In cardiovascular disease, inequities in the quality and type of treatment made available to racial and ethnic minorities have been well documented.^1^ Over a decade ago, Unequal Treatment: Confronting Racial and Ethnic Disparities in Health Care, published by the Institute of Medicine (IOM, 2002^2^), highlighted existing disparities in care. In particular, the report identified the lower rate of necessary cardiovascular procedures—up to 50% lower—performed on black compared to white patients. The IOM findings led to a greater focus on addressing racial and ethnic disparities and initiated more aggressive funding and programming by both public and private organizations.^3^ The quality of cardiovascular care for both blacks and whites has since improved; from 2004 to 2014, cardio-vascular disease (CVD)-attributable death rates for all adults dropped by 25.3% and the actual number of CVD deaths decreased 6.7%.^4^

Together with the decline in mortality, there has been a tightening in the gap between white and black patients on some CVD clinical outcomes, particularly the rates of death attributable to CVD.^5,6^ Significant disparities continue to be evident however. A recent report from the Centers for Disease Control and Prevention indicates that cardiovascular-related mortality has dropped more slowly for black individuals (2.2% per year) than for whites (2.4% per year) for the full study period of 1998–2015. The overall rate of decline for both black and white patients before 2010 (3.4% and 3.9% respectively) decelerated after 2010 and has leveled for both groups to ∼1.0%. Current data demonstrate that the disparity in heart disease death rates is still substantial and shows a 16.3% increase in the black-white mortality ratio from 1968 to 2015.^7^

The continued gap in life expectancy between black and white patients can be largely attributed to cardiovascular-related mortality.^5,8^ This is particularly salient when considering the changing demographics in the United States. The Census Bureau reports that close to 38% of the current population belongs to minority groups, with blacks and Hispanics making up the largest proportions. Census data projects that these populations will continue to grow and predominate by 2045 (https://census.gov/programs-surveys/popproj/data/tables.2017.html). It is, therefore, increasingly critical to focus delivery of care on these populations with CVD.

### What is known about procedure disparities

Decades of research have observed that blacks are less likely to receive beneficial, evidence-based cardiovascular interventions, and have less access to higher-cost invasive procedures, even when controlling for health insurance access. Studies conducted in the 1990s comparing procedure rates for cardiac catheterization, percutaneous coronary intervention (percutaneous transluminal coronary angioplasty [PTCA]), coronary artery bypass graft (CABG), and implantation of defibrillators demonstrated that black patients were less likely to undergo these procedures.^9,10^

More recent studies continue to demonstrate lower rates of cardiac interventions among black patients than white patients.^11^ Cram et al. found that blacks hospitalized with acute myocardial infarction (AMI) were significantly less likely to receive revascularization when compared with whites.^12^ Similarly, Albert et al. identified that the odds of receiving coronary revascularization in black patients is 30% lower than white patients.^13^ Black patients continue to be less likely to receive other effective treatments such as percutaneous coronary intervention, CABG, and implantation of defibrillators.^12,14–16^ A recent study found that blacks were about half as likely to be referred for cardiothoracic surgery as whites.^17^

The differences described are present even when factors such as age, coronary disease severity, comorbid conditions, insurance status, and site of care are controlled.^12,14,16^ A 2014 study of Medicare hospital claims data from 1992 to 2010 found that although differences in procedure rates had declined over time, rates of all procedures (angiography, PCTA, and CABG) were lower for blacks than whites throughout the study period. Even when adjusted for age and comorbidity, racial disparities remained significant.

Other cardiac conditions beyond coronary artery disease also show disparities in evidence-based treatment. Although data on the prevalence of severe aortic stenosis is limited, prevalence of the condition in blacks appears to be lower than for whites (1.51% vs. 0.55%, respectively).^18^ Controlling for this, however, blacks are less likely to receive aortic valve replacement (AVR). A recent study evaluating patients who met the criteria for AVR between 1999 and 2013 demonstrated that a lower proportion of black patients had surgical valve replacement (SAVR) (26.6%) compared with white patients (40.3%).^18^ Similarly, a study evaluating patients diagnosed between 2004 and 2010 found that the proportion of blacks receiving SAVR was significantly lower than for whites (39% vs. 53%).^19^ A small study of 67 patients focused on transcatheter aortic valve replacement (TAVR) found that blacks were significantly less likely to receive TAVR than non-blacks (7% and 92% respectively).^20^ In a recent article, Holmes et al. estimated that in the years 2012–2015, 3.8% of black patients received this therapy compared with 93% in whites.^21^ It is important to note that although blacks are typically higher-risk patients, when treated with AVR, 1 year and 3-year survival has been found to be similar.^18,19^ Importantly, black patients referred for TAVR shared similar risks and outcomes when compared with a white population.^22^

### Study

We analyzed 2012–2013 Medicare data for specific cardiac procedures performed by interventional cardiologists to determine rates of PTCA for AMI and intermediate coronary syndrome (ICS) and rates of TAVR for aortic valve disease (AVD). Our focus on this population of cardiac physicians is intended to isolate the procedures themselves particularly in the acute setting of AMI and ICS and because the procedures occur primarily in a hospital, removing access to ambulatory sites and referring physician networks as a confounding factor. Our focus is on patients who have Medicare fee-for-service as their primary insurance, controlling for the “access to insurance” variable.

### Data

Data from the Medicare 5% Limited Data Set (LDS) files were used in this analysis. The LDS file consists of de-identified beneficiary information in addition to institutional and noninstitutional claims. Institutional claims include claims from inpatient hospital, outpatient facility, skilled-nursing facility, and hospice facility sources. Noninstitutional files include information from physicians. The 5% files do not include outpatient drug claims. Information about patient demographics is contained in the denominator files.

We eliminated patients who did not have Parts A and B coverage in the denominator file for at least 10 months in each year, and members with Health Maintenance Organization (HMO) (Medicare Advantage) coverage. The number of unique members in each year was reduced from an average of 2.7 million beneficiaries to 1.57 million beneficiaries each year due to the selections above. The number of unique members over the period 2012–2013 was ∼1.72 million (Table 1).

**Table 1.** Eligible Population

Sample selections	2012	2013
All members	2,674,799	2,758,260
Exclude: Part A and Part B coverage <10 months/year	(449,725)	(458,563)
Exclude HMO coverage	(658,023)	(719,505)
Final sample (annual)	1,567,051	1,580,192
Final sample—unique members in years 2012–2013	1,715,270	

HMO, Health Maintenance Organization.

## Methods

Our hypothesis is that the procedure rates between races are the same, assuming the same diagnoses and given that all patients have the same insurance coverage. Table 2 shows procedures for three conditions: AMI, ICS, and AVD. Appendix 1 shows the algorithms used to identify patients with different conditions. Note that the number of patients displayed in Table 3 is the number of unique patients with the specific diagnosis in 2012 and 2013. Because individuals entering Medicare are diagnosed and die continuously, we measure the number of months that a patient is observed over the 24-month period with the specific diagnosis. The number of member months is then converted to a number of equivalent member years by dividing by 12. (The same procedure is applied by Centers for Medicare and Medicaid Services (CMS) to calculate equivalent member years—see ResDAC.^23^)

**Table 2.** Procedures for Heart Conditions

Condition	Procedures
Acute myocardial infarction (AMI) or Intermediate coronary syndrome (ICS)	Percutaneous transluminal coronary angioplasty for single major coronary artery
	Percutaneous transluminal coronary angioplasty each additional branch of a major coronary artery branch
	Percutaneous transcatheter placement of intracoronary stent(s), with coronary angioplasty when performed; single major coronary artery or branch for AMI
	Percutaneous transcatheter placement of intracoronary stent(s) for single major coronary artery or branch; each additional branch of a major coronary artery for AMI
Aortic valve disease (AVD)	Transcatheter aortic valve replacement (TAVR) with prosthetic valve; percutaneous femoral artery approach for Aostenosis
	Transcatheter aortic valve replacement (TAVR) with prosthetic valve; transapical exposure (e.g., left thoracotomy) Aostenosi
	Transcatheter aortic valve replacement with prosthetic valve; open axillary artery approach Aostenosis
	Transcatheter aortic valve replacement (TAVR) with prosthetic valve; open iliac artery approach Aostenosis
	Transcatheter aortic valve replacement (TAVR) with prosthetic valve; transaortic approach (e.g., median sternotomy, mediastinotomy) Aostenosis

**Table 3.** Procedure Rates and Costs by Race and by Condition

Race	Conditions	Unique diagnosed members	Procedures	Procedure rate per 1000/year^a^	Procedure cost
Black	AMI	3045	358	68.90^**^	$21,760
White	AMI	25,144	3618	83.03	$19,848
All	AMI	29,251	4217	81.48	$20,090
Black	ICS	2223	256	63.17^**^	$16,151
White	ICS	19,038	2630	73.97	$15,248
All	ICS	22,084	3100	72.90	$15,389
Black	AVD	5339	103	10.57^**^	$49,435
White	AVD	73,479	2643	19.40	$42,329
All	AVD	81,109	2838	18.59	$44,407

^a^ Consistent with CMS, enrollment counts are determined using a person-year methodology.

^**^ Signifies *p*<0.01 level.

CMS, Centers for Medicare and Medicaid Services.

In Table 3 we calculate absolute numbers of procedures for different race/ethnicity groups using the relationship between that race's rate and the white rate per 1000. For example, the procedure rate for PTCA for whites diagnosed with AMI is 83.03 (per 1000 diagnosed patients per year) and that for blacks is 68.90 (per 1000 diagnosed patients per year), resulting in a rate ratio of 1.21 (=83.03/68.90). A ratio greater than 1.00 implies that whites have a 21% higher procedure rate than blacks. The raw procedure count for blacks is 358. To experience an equivalent number of procedures to whites, blacks would require an additional 73 procedures [=358×(1.21 – 1.00)].

## Results

Although we conducted the comparisons between the white population and other races, we focused on comparing two groups—white versus black.

Table 2 shows procedures for three conditions: AMI, ICS, and AVD.

For Acute AMI and ICS conditions, we focused on PTCA. There are four types of such procedures—(i) PTCA for single major coronary artery or branch for AMI, and (ii) PTCA for each additional branch of a major coronary artery, (iii) Placement of intracoronary stents for single major coronary artery or branch, and (iv) Placement of intracoronary stents for each additional branch of a major coronary artery for AMI. (For the codes to identify these procedures see Appendix A2.) We used the same code set for identifying PTCA for the ICS condition.

For AVD, we focused on identifying TAVR procedures: (i) TAVR with prosthetic valve; percutaneous femoral artery approach for AoStenosis, (ii) TAVR with prosthetic valve; open femoral artery approach Aostenosis, and (iii) TAVR with prosthetic valve; open axillary artery approach Aostenosis. (iv) TAVR with prosthetic valve; open iliac artery approach Aostenosis, and (v) TAVR with prosthetic valve; transaortic approach (e.g., median sternotomy, mediastinotomy) Aostenosis.

Procedure rates are based on 1000 diagnosed members per year. For AMI, the white PTCA procedure rate (83.03/1000) is high relative to the black PTCA procedure rate (68.90/1000). A significant difference is also observed for the ICS condition where the procedure rate for whites (73.97/1000) is statistically significantly higher, relative to the black rate (63.17/1000). For the AVD condition we also found that the white procedure rate (19.40/1000) is statistically significantly higher relative to the rates of blacks (10.57/1000).

In Table 3 we sum all relevant procedures (per Table 2) for each diagnosis and report the number of diagnosed patients by race, procedure rates per 1000 diagnosed patients, and the average cost per procedure.

### Financial implications of under-provision of procedures

The financial implications of differences in procedure rates are the work that we are highlighting in this article compared with previous studies that focused on procedure rates only. Table 5 shows the calculation to estimate underservice of procedures and its monetary magnitude. We estimated the under-provision of procedures in other races, assuming that other races experienced procedures at the same rate as the white population. We estimate the under-provision of procedures as:

\begin{align*}
{ \rm{Changes \ in \ procedures}} = &  \big ( {{ \rm{Procedure \
rate \ for \ white \ race}} } \\& /{{ \rm{Procedure \ rate \ black
\ race}}} \big ) \\& \times { \rm{Total \ procedures \ for \ black
\ race}}
\end{align*}

In Table 3 we saw that procedure rates for PTCA for people with AMI was 68.90/1000 for blacks versus 83.03/1000 for whites. If black procedure rates were equal to those of whites, blacks would experience 20.5% more procedures, (358×0.205=73.38 PTCA procedures over the 2-year period). Annually, we estimate an increase in procedures of 73.38/2 or 36.69 additional procedures. Multiplying 36.69 additional procedures by the average cost per black patient procedure in the inpatient setting, $21,760, we estimate reimbursement of the additional procedures as $798,403. Table 5 shows that the result of normalizing the black procedure rate to be equal to that of the white population would increase reimbursement for this procedure by $798,403 per year in 2012–2013 dollars. The table also shows estimates of the increase in revenue due to normalizing the under-provision of services for other two conditions.

As described previously, this analysis was conducted using the 5% sample file of the total Medicare population, excluding the Medicare Advantage (HMO) population. We can estimate the effect of normalizing procedure rates for all of Traditional (fee-for-service) Medicare by grossing up the 5% estimate. We can also make a rough estimate of the effect of normalization on the Medicare Advantage population using the relative Medicare Advantage and Traditional Medicare enrollments from 2012 to 2013. Relative enrollments from CMS reports are shown in Table 4, using data from the CMS Statistics Reports for 2013^24^ and 2014.^25^ Estimated Medicare Advantage enrollment includes prepaid Medicare and Medicare Cost contract enrollment and excludes private fee-for-service, Programs of All-Inclusive Care for the Elderly (PACE), and so on. Average 2012–2013 Medicare Advantage enrollment represented 36.7% of Traditional Medicare enrollment.

**Table 4.** Total Medicare Enrollment 2012–2013

	000,000's	
	Fee for service	Medicare advantage
2012	37.214	13.104
2013	37.587	14.388
Average	37.401	13.746

In Table 5 we estimate the total undertreatment loss, based on evidence-based treatment to be $90 million. Traditional Medicare represents $65.5 million in total and Medicare Advantage a further $24.6 million. These are necessarily estimates although they provide an idea of the financial effects of performing appropriate and life-saving procedures in under-served populations at the white population rate.

**Table 5.** Financial Effect of Normalizing Procedure Rates

					Estimated annual financial effect		
Race	Condition	Total estimated procedures	Change in procedures	Procedure cost	5% Sample	Traditional Medicare	Medicare advantage
Black	AMI	431.38	73.38	$21,760	$798,403	$15,968,062	$6,003,991
Black	ICS	299.74	43.74	$16,151	$353,238	$6,845,743	$2,574,000
Black	AVD	197.14	86.14	$51,147	$2,203,023	$42,653,347	$16,037,658
All					$3,354,664	$65,467,152	$24,615,649

## Discussion: A Plan for Normalizing Care for Minority Communities

The 2016 National Health care Quality and Disparities Report found that “while 20% of measures show disparities getting smaller for blacks and Hispanics, most disparities have not changed significantly for any racial and ethnic groups”^26^ With a focus on cardiac procedural care for blacks, our analysis shows inequities in the rate of cardiovascular procedures. Black patients experience fewer evidence-based treatments than whites, providers and cardiologists and other stakeholders face substantial “lost” opportunity to provide cost-effective care. Though improving clinical outcomes is incentive enough in a value-based world, financial incentives can provide greater impetus. “Normalizing” procedure rates among blacks to that of whites, consistent with their disease prevalence, will require engagement of all stakeholders using data-driven input and targeting measurable solutions.

Solutions that have the potential to help normalize procedure rates and decrease disparities include providing better and more regional and individual provider data, focusing on embedded electronic medical record (EMR) toolsets, and supporting patient understanding of treatment options. Cardiovascular registries that link to health insurance claims and/or EMR data can conceivably provide transparent information about treatment cost and quality for various racial and ethnic groups. These tools can guide physicians in their patient communication and treatment recommendations, improving consistency of appropriate cardiovascular procedures among patient groups.

Advocacy organizations like Association of Black Cardiologists (ABC), American College of Cardiology (ACC), American Hospital Association (AHA), and Cardiovascular Research Foundation (CRF) are positioned to drive awareness and educate both physicians and patients on specific aspects of cardiovascular care through directed programming and communication activities.^27^ Advocacy organizations can use their large member and communication networks to disseminate information and impact measurable change.

### Limitations

This study has a number of limitations. The lower procedure rates demonstrated for black patients could be based on patient preference, which we were not able to ascertain. Race of the patients' cardiologist may impact the treatment decision and we did not have that information for our assessment. Black patients treated by a physician who looks like them may feel a greater sense of trust and have a heightened understanding of the health decisions to be made.^28^ The data presented here are from 2012 to 2013. There may have been improvements in treatment of blacks since that time. Finally, the costs used in the estimates presented here come from the CMS LDS data set; however, it is possible that these costs are not complete and may skew the estimates presented.

## Conclusion

We find that, consistent with other studies, Black patients with cardiac conditions are under-served with respect to evidence-based procedures. We share the lost revenue of undertreatment in parallel, to place financial terms with this clinical cost. Across all Medicare patients we estimate that ∼$90 million in revenue annually is lost by cardiologists as a result of the lower procedure rates. Normalizing care can significantly impact patients' lives today and decrease the long-term cost of more severe, chronic conditions.

## Statement of Informed Consent

This study was a retrospective data analysis using a de-identified, publicly available database (The Medicare Limited Data Set). No informed consent was required.

## Statement of Human and Animal Rights

This study was a retrospective data analysis using a de-identified, publicly available database (The Medicare Limited Data Set). Human and Animal rights are not applicable.

## IRB Waiver

This retrospective study was performed entirely using an administrative claims dataset, the de-indentified Limited Data Set (5% File) from CMS. No human subjects or identifiable data were involved in this study. IRB approval was not required in this circumstance.

## Appendix

### Appendix

**Appendix A1.** Disease Identification Algorithm

Disease algorithms	Reference period (years)	ICD-9/CPT4/HCPCS codes	Number/types of claims to qualify
Acute myocardial infarction (AMI)	1	DX 410.01, 410.11, 410.21, 410.31, 410.41, 410.51, 410.61, 410.71, 410.81, 410.91 (ONLY first or second Dx on the claim)	At least one inpatient claim with Dx code
Aortic valve disease^3^ (AVD)	2	424.1, 396.0, 396.1, 396.2, 396.3, 396.8, 396.9, 746.3, 746.4, 395.0, 395.2, 395.1, and 395.9 (any Dx on the claim)	At least one inpatient, HOP or Carrier claim with Dx code
Intermediate coronary syndrome (ICS)	2	411.1 (any Dx on the claim)	At least 1 inpatient, HOP or Carrier claim with Dx code

Source: CMS Chronic Conditions Data Warehouse (CCW).^29^

ICD-9, International Classification of Diseases, Version 9; CPT4, Current Procedural Terminonlogy, 4th edition; HCPCS, health care common procedure coding system; HOP, hospital outpatient.

### Appendix

**Appendix A2.** Procedure Identification

CPT/HCPCS codes	Description of CPT/HCPCS codes	ICD-9-proc codes	Description of ICD-9-procedure codes	MS-DRG	APC
92920	Percutaneous transluminal coronary angioplasty; single major coronary artery or branch for AMI)	00.66	Percutaneous transluminal coronary angioplasty	250: Percutaneous cardiovascular procedures without coronary artery stent with MCC	83: Coronary angioplasty, valvuloplasty and Level I endovascular Revascularization of the lower extremity
92921	Percutaneous transluminal coronary angioplasty; each additional branch of a major coronary artery (list separately in addition to code for primary procedure) for AMI)	00:66	Same	Same	Same
92928	Percutaneous transcatheter placement of intracoronary stent(s), with coronary angioplasty when performed; single major coronary artery or branch for AMI)	36.06: plus 00.66, 00.40–00.48	Insertion of non-drug-eluting coronary artery stent(s)	248: Percutaneous cardiovascular proc w non-drug-eluting stent with MCC or 4+ vessels/stents 249: Percutaneous cardiovascular proc. w non-drug-eluting stent without MCC	104: Transcatheter placement of Intracoronary stents
92929	Percutaneous transcatheter placement of intracoronary stent(s), with coronary angioplasty when performed; each additional branch of a major coronary artery (list separately in addition to code for primary procedure) for AMI)	Same	Same	Same	Same
33361	Transcatheter aortic valve replacement (tavr/tavi) with prosthetic valve; percutaneous femoral artery approach for AoStenosis	35.05 35.09	Endovascular replacement of aortic valve, Endovascular replacement of unspecified heart valve	216–221, 237–238	
33362	Transcatheter aortic valve replacement (tavr/tavi) with prosthetic valve; open femoral artery approach Aostenosis	Same	Same	Same	
33363	Transcatheter aortic valve replacement (tavr/tavi) with prosthetic valve; open axillary artery approach Aostenosis	Same	Same	Same	
33364	Transcatheter aortic valve replacement (tavr/tavi) with prosthetic valve; open iliac artery approach Aostenosis	Same	Same	Same	
33365	Transcatheter aortic valve replacement (tavr/tavi) with prosthetic valve; transaortic approach (e.g., median sternotomy, mediastinotomy) Aostenosis	Same	Same	Same	

Source: Boston Scientific.^30^

MS-DRG, Medicare Severity Diagnosis Related Group; APC, ambulatory payment classification; MCC, major complications or co-morbidities.

## Abbreviations Used

ABC: Association of Black Cardiologists
ACC: American College of Cardiology
AHA: American Hospital Association
AMI: acute myocardial infarction
APC: ambulatory payment classification
AVD: aortic valve disease
AVR: aortic valve replacement
CMS: Centers for Medicare and Medicaid Services
CPT-4: Current Procedural Terminology, 4th Edition
CRF: Cardiovascular Research Foundation
CVD: cardio-vascular disease
EMR: electronic medical record
HCPCS: healthcare common procedure coding system
HMO: Health Maintenance Organization
HOP: hospital outpatient
ICD-9: International Classification of Diseases, Version 9
ICS: intermediate coronary syndrome
IOM: Institute of Medicine
LDS: limited data set
MCC: major complications or co-morbidities
MS-DRG: Medicare severity diagnosis related group
PCI: percutaneous coronary intervention
PTCA: percutaneous transluminal coronary angioplasty
SAVR: surgical valve replacement
